# 1665. Potential underreporting of treated patients using a *Clostridioides difficile* testing algorithm that screens with a nucleic acid amplification test

**DOI:** 10.1093/ofid/ofac492.131

**Published:** 2022-12-15

**Authors:** Alice Guh, Lisa Gail Winston, Helen Johnston, Elizabeth Basiliere, Danyel M Olson, Scott Fridkin, Dana Goodenough, Christopher Wilson, Jasmine Watkins, Lauren C Korhonen, Dale N Gerding

**Affiliations:** CDC, Atlanta, GA; UCSF, San Francisco, California; Colorado Department of Public Health, Denver, Colorado; Colorado Department of Public Health and Environment, Denver, Colorado; Yale School of Public Health, New Haven, Connecticut; Emory University School of Medicine, Atlanta, GA; Emory University School of Medicine, Atlanta, GA; Tennessee Department of Health, Nashville, Tennessee; Tennessee Department of Health, Nashville, Tennessee; CDC, Atlanta, GA; Edward Hines, Jr. Veterans Affairs Hospital, Hines, Illinois

## Abstract

**Background:**

U.S. laboratories are increasingly using a two-step algorithm to diagnose *Clostridioides difficile* infection (CDI) that starts with a nucleic acid amplification test (NAAT), and if positive, reflexes to a toxin enzyme immunoassay. Here only a NAAT+/toxin+ result is reported to the Centers for Disease Control and Prevention’s (CDC) National Healthcare Safety Network (NHSN) as a CDI laboratory-identified (LabID) event, but limited data suggest that NAAT+/toxin- results may also be considered CDI by clinicians. To explore this discrepancy, we compared the characteristics and treatment of NAAT+/toxin- and NAAT+/toxin+ patients.

**Methods:**

CDC’s Emerging Infections Program (EIP) conducts population-based CDI surveillance. A case was defined as a positive *C. difficile* test in a person aged ≥1 year with no positive tests in the prior 8 weeks. We included cases detected by this two-step algorithm in 5 EIP sites during 2018–2020 that underwent a full chart review. Multivariable logistic regression models adjusting for age, sex, race, comorbidities, epidemiologic classification, and CDI therapy were used to compare CDI-related complications (i.e., toxic megacolon, ileus, colectomy, or intensive-care unit stay) and recurrence between the two groups.

**Results:**

Of 1250 NAAT+ cases, 897 (72%) were toxin- and 353 (28%) were toxin+. Lower percentages of toxin- versus toxin+ cases were aged ≥65 years (42% vs 58%; P< 0.0001), had diarrhea (779/831 [94%] vs 329/338 [97%]; P=0.01), or had white blood cell counts ≥15,000 cells/μL (183/811 [23%] vs 132/321 [42%]; P< 0.0001). CDI therapy was given to 683/882 (77%) toxin- versus 338/349 (97%) toxin+ cases (P< 0.0001). In multivariable analysis, toxin- status was protective for recurrence (adjusted odds ratio [aOR], 0.49; 95% confidence interval [CI], 0.32–0.74) but not for CDI-related complications (aOR, 1.00; 95% CI, 0.64–1.56).

**Conclusion:**

NAAT+/toxin- cases were less likely to have recurrence but were as likely to have CDI-related complications as NAAT+/toxin+ cases. More than twice as many potentially unreported NAAT+/toxin- cases were treated than the number of reported NAAT+/toxin+ treated cases. Use of this two-step algorithm likely results in underreporting of treated CDI cases to NHSN.

**Disclosures:**

**Scott Fridkin, MD**, Pfizer: Grant/Research Support **Dale N. Gerding, MD**, Destiny Pharma plc.: Advisor/Consultant.

